# Incidental Intramyocardial Bridging in a Myocarditis Patient Presenting With Focal ST Segment Depressions

**DOI:** 10.7759/cureus.9931

**Published:** 2020-08-21

**Authors:** Kriti Gupta, Asiya Batool, Arsalan Talib Hashmi, Michael Marcelin

**Affiliations:** 1 Medicine, Maimonides Medical Center, Brooklyn, USA; 2 Internal Medicine, Jinnah Hospital Lahore (JHL)/Allama Iqbal Medical College (AIMC), Lahore, PAK; 3 Cardiology, Maimonides Medical Center, Brooklyn, USA; 4 Internal Medicine, Maimonides Medical Center, Brooklyn, USA

**Keywords:** myocarditis, lv strain, intramyocardial bridging, st changes, acute cardiac care, cardiac risk factors and prevention, coronary vessel anomaly, cardiac catheterization

## Abstract

Myopericarditis is an entity known to present with typical symptoms of viral prodrome and diffuse ST elevation (STE) and/or PR depressions on electrocardiogram (EKG). Atypical presentations of myocarditis such as focal STE have been cited in the literature, reflecting true coronary ischemia. However, myocarditis or pericarditis presenting with focal ST depressions is rarely seen. Myocarditis is usually overlooked as a differential for ST depressions seen on EKGs; hence, the case we present in this report highlights the importance of nonischemic causes presenting as ischemic changes on EKG. This case is unique as we have postulated a possible explanation for this finding.

This report discusses the case of a young patient with myopericarditis presenting with focal ST depressions. This patient was also incidentally found to have intramyocardial bridging, usually a benign finding, on cardiac catheterization (which is shown in the case report). Our hypothesis is that the inflammation due to myocarditis in this patient led to inflammation of intramyocardial vessels, presenting as ST depressions. Since intramyocardial bridging is a common anomaly, we propose the question as to whether this could be a risk factor for sudden cardiac death and if it depends on the characteristic of the intramyocardial vessel. We would like to emphasize on the atypical presentations of this usual condition, a possible explanation for this finding, and the need for further testing for risk stratification in patients with this anomaly.

## Introduction

Myopericarditis is a rare entity, and it is defined as the inflammation of the myocardium and pericardium usually secondary to a viral syndrome. It presents with a viral prodrome and cardiac symptoms, most commonly diffuse ST elevations (STE) and/or PR segment depressions on electrocardiogram (EKG) [1]. There has been an increasing number of cases in the literature citing the presence of focal STE in cases of myocarditis and myopericarditis [2-10]. Ischemia in these cases is thought to be secondary to vasospasm [2]. This pattern of severe vasospasm mimicking acute myocardial infarction is most commonly seen in patients with biopsy-proven parvovirus B19-induced myocarditis [2]. However, the presence of ST depression is an extremely rare finding in patients diagnosed with myopericarditis [3].

Intramyocardial bridging, a congenital anomaly in which an epicardial coronary artery takes an intramyocardial course, is a common anatomical anomaly that usually has no clinical significance [11]. In rare cases, unexplained ST segment elevations have been hypothesized to occur due to this anomaly, and the extent of intramyocardial bridging may even contribute to morbidity secondary to coronary diseases [12]. The few cases of acute coronary syndrome reported in patients with intramyocardial bridging are, on the other hand, thought to be due to accelerated atherosclerosis [12]. In this report, we present the case of a 29-year-old healthy male with chest pain and ST depressions on presentation EKG; there was no evidence of coronary artery disease (CAD) on angiography, but severe intramyocardial bridging was found. This patient was subsequently diagnosed with myopericarditis on cardiac magnetic resonance imaging (CMRI). We discuss the possible mechanisms and significance of this unusual case.

## Case presentation

A 29-year-old Caucasian male with no past medical history presented to a community hospital in Brooklyn, United States, with chest pain of one day's duration. He reported that the chest pain had begun acutely earlier on the day of the admission when he had gone up a flight of stairs. He described a pressure-like chest pain that was 8/10 in severity, which did not radiate, was not reproducible to palpation, improved on rest, and with no change inspiration. He denied nausea, vomiting, shortness of breath, or diaphoresis. He reported experiencing similar pain the day prior to the admission that had lasted for two hours and had occurred on rest. As the chest pain had not remitted, he had presented to an urgent care center and had been found to have diffuse ST changes including STE in the posterior leads with ST depressions; he had been subsequently referred to our emergency department (ED). He endorsed having a diarrheal illness that had started about seven days before the onset of chest pain. He denied the use of any drugs.

**Figure 1:**
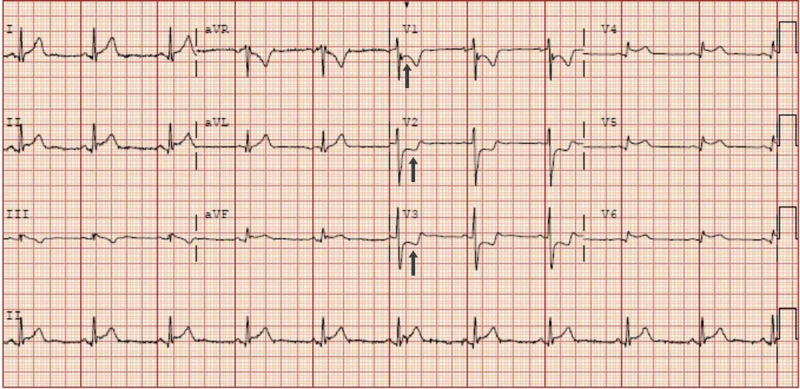
Presentation EKG of the patient The arrows show ST segment depression in leads V1, V2, and V3 EKG: electrocardiogram

Vital signs measured in the ED were as follows: heart rate of 61 beats per minute, blood pressure of 130/68 mmHg, respiratory rate of 16 breaths per minute, and oxygen saturation of 100% on room air. He was afebrile and found to have regular rate and rhythm with no murmurs on physical exam. Initial baseline blood work included complete blood count, arterial blood gas, and basic metabolic panel, which were all within normal limits. A urine toxicology screen was negative. Troponin I levels on presentation was 8.3 ng/ml. Other laboratory results including a complete blood count and a comprehensive metabolic profile were within normal limits. EKG at the time of presentation showed ST segment elevation in leads V4 and V5 and ST segment depression in leads V1, V2. and V3 (Figure 1).

The patient was taken for emergency cardiac catheterization. The findings on coronary angiogram performed via right radial access revealed a normal left main artery, left circumflex artery, right coronary artery, and left anterior descending artery (LAD). However, there was also severe intramyocardial bridging of the LAD. Video 1 demonstrates imaging during cardiac catheterization showing severe intramyocardial bridging of the LAD.

**Video 1:** Cardiac catheterization The arrow shows intramyocardial bridging of the LAD LAD: left anterior descending artery

**Figure 2:**
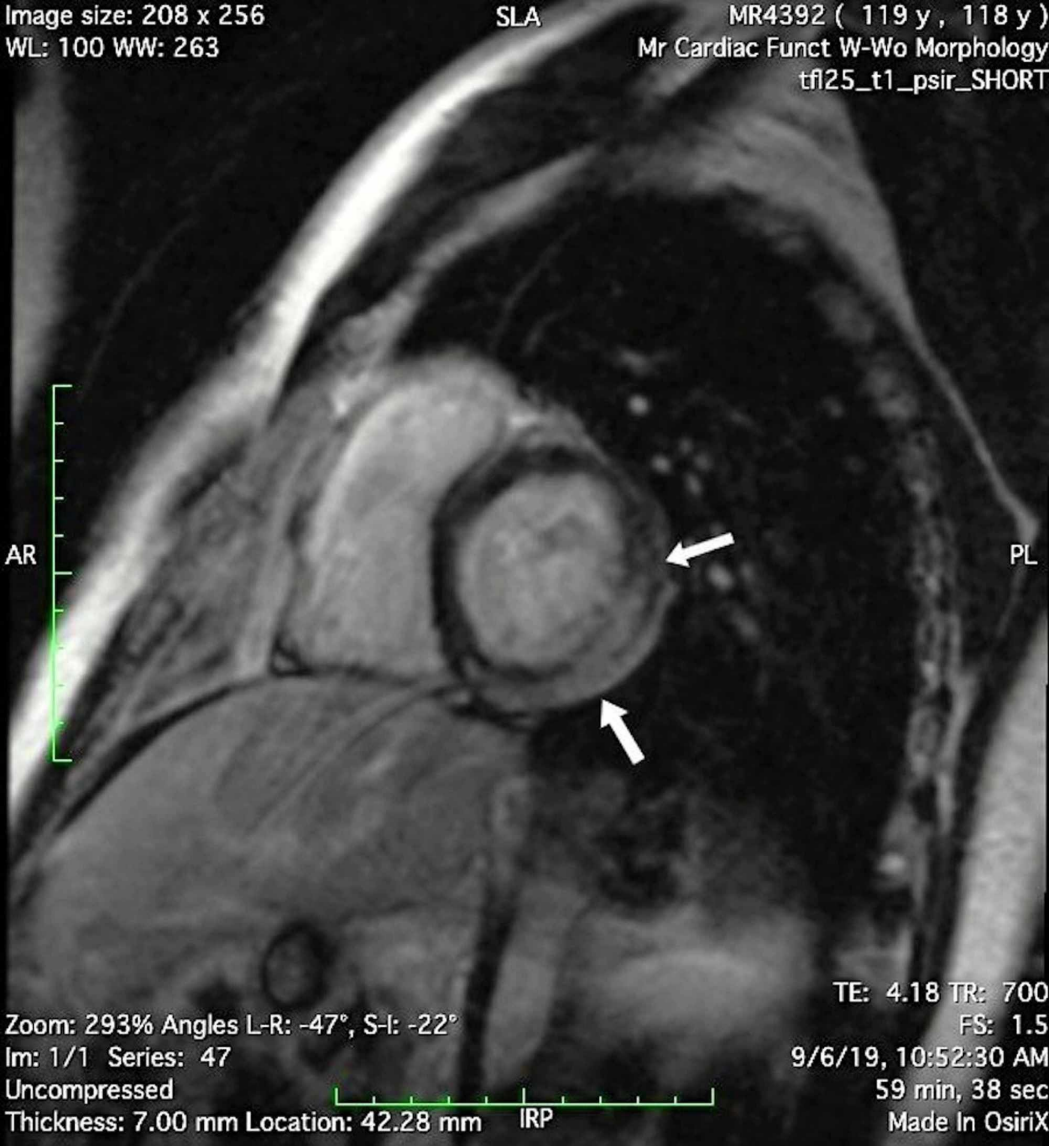
CMRI of the patient The arrows show delayed enhancement of the midmyocardium, epicardium, and adjacent pericardium at the mid-cavity level of the left ventricle. This has been interpreted as extensive myocarditis/pericarditis in the inferior and lateral wall of the left ventricle CMRI: cardiac magnetic resonance imaging

An echocardiogram showed wall motion abnormalities of the entire inferior wall, basal and mid anterolateral wall, and basal and mid inferolateral, and an ejection fraction of 40-45%. He further underwent CMRI with gadolinium contrast, which revealed extensive myocarditis/pericarditis in the inferior and lateral wall of the left ventricle. CMRI further revealed extensive delayed enhancement of inferior and lateral wall, with a delayed enhancement of epicardium and adjacent pericardium at the base of the left ventricle, delayed enhancement of the midmyocardium, epicardium, and adjacent pericardium at the mid-cavity level of the left ventricle, and delayed enhancement of epicardium and adjacent pericardium at the apical level of the left ventricle (Figure 2).

The patient was treated supportively with intravenous fluids, and nonsteroidal anti-inflammatory drugs were given for pain control. His symptoms improved and troponin levels trended down to 0.03. He was safely discharged home.

## Discussion

The term myopericarditis primarily denotes a pericarditic syndrome associated with inflammation of the myocardium. Often, these two coexist due to the common etiological factors, mainly cardiotropic virus. It is difficult to determine the true incidence of myopericarditis as it is likely that a significant number of cases go unnoticed due to their asymptomatic nature [13]. A study by Sharma et al. in 2015 estimated that myopericarditis accounts for less than 2% of inpatient admissions for chest pain [6]. Due to the variability of myocardial involvement, the clinical presentation is varied, with overlapping features of viral syndrome and cardiac symptoms. As there is a lack of a true standard criterion for diagnosing pericarditis and myocarditis, it is often difficult to differentiate it from acute myocardial infarction [14,15]. The diagnostic criteria published by the European Heart Journal in 2013 involve new electrocardiographic findings including STE, non-ST elevation, and T wave inversion [16].

Our patient, a young male, clinically did not have any risk factors for coronary atherosclerosis and presented with only chest pain. This brought up suspicion for cardiac chest pain unrelated to coronary pathology. However, the presence of ST segment depression on presentation EKG raised the differential of acute coronary syndrome. Given the absence of any evidence of CAD on angiography, other causes of cardiac chest pain were pursued. It is estimated that approximately 10% of cases of suspected CAD are found to have normal coronary arteries on angiography [17], and this warrants the workup for an alternative diagnosis; hence, the possibility of myocarditis or pericarditis was considered. We subsequently performed a CMRI. On a CMRI, myocarditis shows a characteristic pattern of contrast enhancement, which originates primarily from the epicardium, sparing the subendocardial layer, which was evident in our patient [18,19].

A significant number of cases of myocarditis with focal ST segment elevations have been reported in the literature [1-10]. To our knowledge, there are very few case reports of myopericarditis presenting as ST depression. According to a case series study of 34 cases of myocarditis by Dec et al., only two cases had ST depression [1]. Focal STE with reciprocal changes as reported by Yildirim et al. [4], STE in an adolescent male reported by Sharma et al. [6], and STE in the inferior leads in a 15-year-old male as reported by Nisbet et al. [9] highlight the pattern of EKG changes in this condition. Sharif et al. compared two cases with a similar clinical presentation with STE, but one with normal coronary anatomy and myopericarditis on CMRI and the other with 90% LAD occlusion [10]. This clearly summarizes the conundrum in differentiating the two. The suspected mechanism of ST depression in myopericarditis is vasospasm caused by myocarditis. Increases in oxidative stress, reduced bioavailability of vasodilator nitric oxide, and ensuing endothelial dysfunction have been implicated as possible mechanisms for the induction of vasospasm [18].

We would like to address the clinical significance of myocardial bridging in this patient. The reported incidence of myocardial bridging is approximately 25% based on autopsies and CT scans performed [11]. Although there have been case reports of myocardial bridging presenting as acute coronary syndrome, no definite correlation between the presence of this finding and the occurrence of ischemia has been established. The pathophysiology in these cases is believed to be due to accelerated atherosclerosis [12]. However, in our case, given the diagnosis of myopericarditis on CMRI and no evidence of CAD on angiography, it is likely that inflammation of myocardium could contribute to ischemia secondary to inflammation of intramyocardial vessels, presenting as ST depressions. A case report from Italy investigated the significance of the presence of myocardial bridging in an athlete who was found to have asymptomatic ST segment changes during sports training [19]. They recommended performing coronary computerized tomography angiogram (CCTA) in patients with low coronary artery disease risk but still presenting with ST segment changes. The suggested purpose of a CCTA would be to identify the presence of a muscular bridge and its length and depth as these characteristics could potentially help in risk stratification. There has been one other case of myocarditis with an incidental finding of myocardial bridging in a young athlete, which was described by Quaranta et al. in the World Journal of Cardiology in 2015. In that case, interestingly, the patient presented with diffuse T wave inversions as opposed to ST depressions seen in our patient [20].

## Conclusions

Very few case reports have cited myopericarditis presenting as ischemic changes, specifically ST depressions on EKG. Besides drawing attention to this disease presenting atypically, the possible mechanism for this presentation has been a conundrum. This case report discussed a case of myopericarditis presenting with focal ST depressions, which was incidentally found to have intramyocardial bridging on CMRI. This led us to a hypothesis that inflammation of the incidentally present intramyocardial vessels in myocarditis patients can reflect as ischemic changes on EKG. The purpose of this case report is to acknowledge the presence of unlikely EKG findings in cases of myopericarditis, and the consideration of this syndrome as a differential of ST depressions. Further studies are required to investigate if there is a link between the length and depth of anomalous coronary artery muscular bridge and the risk of sudden cardiac death. If so, further testing such as CCTA should be strongly considered in patients with low risk of CAD presenting with atypical ST changes for risk stratification.
